# Linguistic Multi-Attribute Group Decision Making with Risk Preferences and Its Use in Low-Carbon Tourism Destination Selection

**DOI:** 10.3390/ijerph14091078

**Published:** 2017-09-17

**Authors:** Hui Lin, Zhou-Jing Wang

**Affiliations:** School of Information, Zhejiang University of Finance and Economics, Hangzhou 310018, China; hhlin0731@163.com

**Keywords:** low-carbon tourism destination selection, linguistic multi-attribute group decision making, risk preference, incomplete weight information, linear program

## Abstract

Low-carbon tourism plays an important role in carbon emission reduction and environmental protection. Low-carbon tourism destination selection often involves multiple conflicting and incommensurate attributes or criteria and can be modelled as a multi-attribute decision-making problem. This paper develops a framework to solve multi-attribute group decision-making problems, where attribute evaluation values are provided as linguistic terms and the attribute weight information is incomplete. In order to obtain a group risk preference captured by a linguistic term set with triangular fuzzy semantic information, a nonlinear programming model is established on the basis of individual risk preferences. We first convert individual linguistic-term-based decision matrices to their respective triangular fuzzy decision matrices, which are then aggregated into a group triangular fuzzy decision matrix. Based on this group decision matrix and the incomplete attribute weight information, a linear program is developed to find an optimal attribute weight vector. A detailed procedure is devised for tackling linguistic multi-attribute group decision making problems. A low-carbon tourism destination selection case study is offered to illustrate how to use the developed group decision-making model in practice.

## 1. Introduction

Climate change caused by carbon emissions has resulted in global warming and created an increasing threat to the environment and survival of all living things on earth. To cope with this challenge, the Chinese government declared at the 2009 United Nations Climate Change Conference its goal to reduce the intensity of carbon emission by 40–45% below 2005 levels, by 2020. A low-carbon economy has been considered to be an effective development framework for carbon reduction and environmental protection without affecting economic enhancement [1,2]. As a significant part of economic development, the tourism industry is encouraging low-carbon tourism and developing low-carbon tourism destinations (LCTDs) [3,4]. Meanwhile, more and more tourists are paying attention to carbon reduction and environmental protection, and thus select low-carbon tourism destinations to relieve the mental pressure caused by their work. Therefore, in order to obtain a high-quality travel experience, it is important for tourists to select the best option(s) from multiple low-carbon tourism destinations based on multi-attributes while considering carbon reduction, lower energy consumption and environmental protection. Generally speaking, tourism destination selection often involves multiple tourists. Each tourist may have his/her own demands and may approach the selection process with different expectations, but all of these tourists have a mutual interest in reaching final agreement on selecting the best travel destination(s). On the other hand, it is difficult for the tourist group to create a ranking order of all possible tourism destinations due to the fact that the multiple attributes or criteria are frequently conflicting. To address such problems in selecting tourism destinations, this paper develops an approach to solve group decision-making problems, where evaluations of all of the alternatives with respect to each attribute are provided as linguistic terms, and the attribute weights are partly known.

Linguistic multi-attribute group decision making (MAGDM) uses linguistic terms in a linguistic term set (LTS) to express decision makers’ evaluations of alternatives with respect to each attribute. In order to aggregate such evaluation values into an overall value in the linguistic environment, Herrera and Martínez [5] developed a 2-tuple linguistic representation model, in which a linguistic term in a balanced LTS is expressed by a linguistic 2-tuple. Wang and Hao [6] devised another 2-tuple linguistic representation model, where trapezoidal fuzzy numbers are used to characterize semantic information of linguistic terms in an unbalanced LTS. Based on these two representation models, a number of aggregation operators have been developed to solve linguistic MAGDM problems, such as the weighted averaging operator [5], the linguistic power aggregation operator [7], the linguistic hybrid harmonic operator [8] and the linguistic Choquet aggregation operator [9]. However, attribute weight information may be incomplete because of the complexity and indeterminacy of MAGDM problems [10,11]. Some researchers have focused their attention on linguistic MAGDM with incomplete weight information. For instance, Wei [12] developed another method to solve linguistic MAGDM problems, where linguistic terms are transformed into 2-tuples and TOPSIS (technique for order performance by similarity to ideal solution [13]) is used to devise an optimization model for determining attribute weights. Wei [14] proposed an approach to MAGDM, in which a maximizing-deviation-based optimization model is established to obtain attribute weights. Zhang and Guo [15] put forward an approach to linguistic MAGDM with multi-granularity and incomplete attribute weight information. By using the positive and negative ideal solutions, Ju [16] presented a method for solving linguistic MAGDM problems with incomplete linguistic weight information. In recent years, MAGDM with linguistic information has been widely used in many different areas, such as company performance assessment [17], recommender systems [18] and supplier selection [19].

Decision-making methods have been applied in low-carbon economy development. Tong and Wang [20] proposed a group decision-making framework with intuitionistic fuzzy preference relations and applied it to low-carbon supplier selection. Cho et al. [3] adopted the fuzzy analytic hierarchy process to construct evaluation indicators of Taiwan’s low-carbon tourism development. Cheng [3] used the Delphi method and the analytic hierarchy process to establish evaluation indicators of low-carbon tourist attractions. Zhang [21] employed the analytic network process to evaluate regional low-carbon tourism strategies. 

In real-life linguistic MAGDM problems, different decision makers often have various expectations and considerations for semantic scale values of linguistic terms, which can be characterized by their risk preferences [22,23,24]. For example, if linguistic terms are used to describe the evaluation of a tourism destination’s low-carbon facilities, then different tourists may have various expectative scale values for the linguistic term “Good”. Zhou and Xu [23] introduced two parameters reflecting risk preferences to extend the sigmoid function and proposed the notion of generalized linguistic term sets (GLTSs). Lin and Wang [24] developed GLTSs with triangular fuzzy semantic information and put forward an approach to solve qualitative decision-making problems. In this paper, we establish an optimization model to obtain an optimal group GLTS based on individual decision maker’s risk preferences. By using the triangular fuzzy weighted average based aggregation method, individual linguistic evaluations are fused into group triangular fuzzy evaluations. Based on the group evaluation information, a linear program is established to obtain optimal attribute weights. A detailed procedure is developed to solve linguistic MAGDM problems with risk preferences and incomplete weight information. 

The remaining contents of this article are organized as follows. The next section gives preliminaries on LTSs, GLTSs and the Euclidean distance of any two positive triangular fuzzy numbers. Section 3 describes linguistic MAGDM problems and establishes a nonlinear programming model to obtain a group GLTS. A linear program and a procedure are developed for solving linguistic MAGDM problems with risk preferences and incomplete weight information in Section 4. Section 5 provides a case study of a low-carbon tourism destination selection problem in order to examine the proposed decision models. Finally, Section 6 offers concluding remarks.

## 2. Preliminaries

This section offers preliminaries on LTSs, GLTSs and the Euclidean distance between two positive triangular fuzzy numbers.

Let $S=\left\{s_{-\tau_{1}},s_{-\tau_{1}+1},\ldots ,s_{0},\ldots ,s_{\tau_{2}-1},s_{\tau_{2}}\right\}$ be an LTS, where $\tau_{1}$ and $\tau_{2}$ are two positive integers, $\tau_{1}+\tau_{2}+1$ is the granularity of S, and *s*_0_ is the neutral linguistic term in S, such as “middle”, “fair” and “indifference”. If $\tau_{1}=\tau_{2}$, then S satisfies the following characteristics [25]:

(i) The set S is ordered, i.e., $s_{i}{}^{}>s_{j}{}^{}$ if and only if i>j;
(ii) A negation operator can be defined as $Neg(s_{i})=s_{-i}$, where $N\text{eg}\left(s_{0}\right)$$=s_{0}$.

A LTS S is called a balanced LTS if $\tau_{1}=\tau_{2}$ and the distribution of its semantic information is uniform and symmetrical; otherwise, S is an unbalanced LTS. For example, an LTS including seven linguistic terms ($\tau_{1}=\tau_{2}=3$) is expressed as:

(1) $$S=\left\{\begin{array}{l} s_{-3}=very\;poor(VP),s_{-2}=poor(P),s_{-1}=slightly\;poor(SP),s_{0}=medium(M),\\ s_{1}=slightly\;good(SG),s_{2}=good(G),s_{3}=very\;good(VG) \end{array}\right\}$$

In order to characterize semantic information with risk preferences for linguistic terms in a LTS, Lin and Wang [24] introduced the following notion of a GLTS.

**Definition 1 [24].**  *For an LTS* $S=\{s_{-\tau_{1}},s_{-\tau_{1}+1},\ldots ,s_{0},\ldots ,s_{\tau_{2}-1},s_{\tau_{2}}\}$*, a GLTS is defined as*

(2) $$\tilde{S}=\left\{\langle s_{i},{\tilde{v}}_{i}\rangle |i=-\tau_{1},\ldots ,0,\ldots ,\tau_{2},{\tilde{v}}_{i}=(v_{i}^{L},v_{i}^{M},v_{i}^{U})\right\}$$

*where* ${\tilde{v}}_{i}$ *is a triangular fuzzy number with two parameters* $\theta_{1}$ *and* $\theta_{2}$ ($\theta_{1},\theta_{2}>0$) *and indicates a fuzzy semantic value of *$s_{i}$ *for each* $i=-\tau_{1},\ldots ,0,\ldots ,\tau_{2}$*, and* $v_{i}^{L},v_{i}^{M}$ *and* $v_{i}^{U}$ *are given as*

(3) $$v_{i}^{L}=\{\begin{array}{ll} {\left(1+e^{-\theta_{2}i}\right)}^{-1}, & i=-\tau_{1}\\ {\left(1+e^{-\theta_{2}(i-1)}\right)}^{-1}, & i=-\tau_{1}+1,\ldots ,-1,0\\ {\left(1+e^{-\theta_{1}(i-1)}\right)}^{-1}, & i=1,2,\ldots ,\tau_{2} \end{array}$$

(4) $$v_{i}^{M}=\{\begin{array}{ll} {\left(1+e^{-\theta_{2}i}\right)}^{-1}, & i=-\tau_{1},\ldots ,-1\\ 0.5, & i=0\\ {\left(1+e^{-\theta_{1}i}\right)}^{-1}, & i=1,2,\ldots ,\tau_{2} \end{array}$$

(5) $$v_{i}^{U}=\{\begin{array}{ll} {\left(1+e^{-\theta_{2}(i+1)}\right)}^{-1}, & i=-\tau_{1},\ldots ,-1\\ {\left(1+e^{-\theta_{1}(i+1)}\right)}^{-1}, & i=0,1,\ldots ,\tau_{2}-1\\ {\left(1+e^{-\theta_{1}i}\right)}^{-1},\; & i=\tau_{2} \end{array}$$

It is obvious that $v_{-1}^{U}=v_{0}^{M}=v_{1}^{L}=0.5$ and $0<v_{i}^{L},v_{i}^{M},v_{i}^{U}<1$ for $i=-\tau_{1},\ldots ,0,\ldots ,\tau_{2}$. If $\theta_{1}>\theta_{2}$, then the risk preferences of semantic values of linguistic terms in $\tilde{S}$ are radical. If $\theta_{1}=\theta_{2}$, then the risk preferences of semantic values of linguistic terms in $\tilde{S}$ are neutral. If $\theta_{1}<\theta_{2}$, then the risk preferences of semantic values of linguistic terms in $\tilde{S}$ are aversive.

For any two positive triangular fuzzy numbers ${\tilde{v}}_{\alpha }=(v_{\alpha }^{L},v_{\alpha }^{M},v_{\alpha }^{U})$ and ${\tilde{v}}_{\beta }=(v_{\beta }^{L},v_{\beta }^{M},v_{\beta }^{U})$, their Euclidean distance is given as [26]

(6) $$d({\tilde{v}}_{\alpha },{\tilde{v}}_{\beta })=\sqrt{\frac{1}{3}\left({\left(v_{\alpha }^{L}-v_{\beta }^{L}\right)}^{2}+{\left(v_{\alpha }^{M}-v_{\beta }^{M}\right)}^{2}+{\left(v_{\alpha }^{U}-v_{\beta }^{U}\right)}^{2}\right)}$$

It is obvious that $0\leq d({\tilde{v}}_{\alpha },{\tilde{v}}_{\beta })<1$ if $0<v_{\alpha }^{L}\leq v_{\alpha }^{M}\leq v_{\alpha }^{U}\leq 1$ and $0<v_{\beta }^{L}\leq v_{\beta }^{M}\leq v_{\beta }^{U}\leq 1$. According to (2)–(6), we have $d({\tilde{v}}_{-i}^{},{\tilde{v}}_{0}^{})=d({\tilde{v}}_{i}^{},{\tilde{v}}_{0}^{})$ $\left(i=1,2,\ldots ,\tau_{2}\right)$ if $\tau_{1}=\tau_{2}>1$ and $\theta_{1}=\theta_{2}$. This implies that the distribution of semantic values of linguistic terms in $\tilde{S}$ is symmetrical. In this situation, if $d({\tilde{v}}_{i}^{},{\tilde{v}}_{i+1}^{})$ is approximately equal to $d({\tilde{v}}_{i-1}^{},{\tilde{v}}_{i}^{})$, i.e., $d({\tilde{v}}_{i}^{},{\tilde{v}}_{i+1}^{})\approx d({\tilde{v}}_{i-1}^{},{\tilde{v}}_{i}^{})$ for $i=-\tau_{1}+1,\;\ldots ,\;-1,\;0,\;1,\;\ldots ,\;\tau_{2}-1$, then $\tilde{S}$ is said to have symmetry and approximate uniformity; otherwise, $\tilde{S}$ is symmetrical and non-uniform. 

If $\tau_{1}=\tau_{2}>1$ and $\theta_{1}\neq \theta_{2}$, then one has $d({\tilde{v}}_{-i}^{},{\tilde{v}}_{0}^{})\neq d({\tilde{v}}_{i}^{},{\tilde{v}}_{0}^{})$$\left(i=1,2,\ldots ,\tau_{2}\right)$ and there exists $i\in \{-\tau_{1}+1,\ldots ,-1,0,1,\ldots ,\tau_{2}-1\}$ satisfying $d({\tilde{v}}_{i}^{},{\tilde{v}}_{i+1}^{})\neq d({\tilde{v}}_{i-1}^{},{\tilde{v}}_{i}^{})$. This indicates that the distribution of semantic values of linguistic terms in $\tilde{S}$ is asymmetrical and non-uniform.

In order to compare and rank triangular fuzzy numbers, the following formula is used to obtain the score of a triangular fuzzy number $\tilde{v}=(v_{}^{L},v_{}^{M},v_{}^{U})$ [27].

(7) $$S(\tilde{v})=\frac{v_{}^{L}+2v_{}^{M}+v_{}^{U}}{4}$$

## 3. An Optimization Model for Determining a Group Generalized Linguistic Term Set

This section describes an MAGDM problem and establishes an optimization model to obtain an optimal group GLTS.

Given n feasible alternatives $x_{i}(i=1,2,\ldots ,n)$ and *m* qualitative attributes $a_{j}(j=1,2,\ldots ,m)$. Let $X=\text{\{}x_{1},\;x_{2},\ldots ,\;x_{n}\text{\}}$ be the alternative set and $A=\text{\{}a_{1},\;a_{2},\ldots ,\;a_{m}\text{\}}$ be the attribute set, then an MAGDM problem is to determine a ranking of all alternatives or find the best alternative(s) from feasible alternatives in X according to the evaluation information offered by a group of experts or decision makers denoted by $E=\text{\{}e_{1},\;e_{2},\ldots ,\;e_{q}\text{\}}$.

Assume that the important weight vector of the experts is $\lambda ={\left(\lambda_{1},\lambda_{2},\ldots ,\lambda_{q}\right)}^{T}$, where $0\leq \lambda_{k}\leq 1$ for each k=1,2,…,q, and the weight vector is normalized to one, i.e., $\sum_{k=1}^{q}\lambda_{k}=1$. In linguistic MAGDM, each expert $e_{k}\in E$ uses linguistic terms in S to evaluate the alternatives in *X* with respect to the attributes in A and provides a decision matrix as $R_{k}={\left(s_{r_{ijk}}\right)}_{n\times m}$, where $s_{r_{ijk}}$ is a linguistic term in S, i.e., $s_{r_{ijk}}\in S$ for i=1,2,…,n,j=1,2,…,m,k=1,2,…,q. 

In linguistic MAGDM with risk preferences, different experts have various expectations and considerations for the semantic scale value of a linguistic term. In other words, different decision makers have various risk preferences on providing their evaluation information. In order to aggregate individual evaluations into a group evaluation, it is necessary to determine a group GLTS based on the expected triangular fuzzy semantic values given by the decision maker $e_{k}\in E,k=1,2,\ldots ,q$ for linguistic terms in S.

Let ${\tilde{V}}^{\left(k\right)}=\left\{{\tilde{v}}_{t_{\alpha }}^{\left(k\right)},{\tilde{v}}_{t_{\beta }}^{\left(k\right)},\ldots ,{\tilde{v}}_{t_{\gamma }}^{\left(k\right)}\right\}$ be the set of the expected triangular fuzzy semantic information provided by the decision maker $e_{k}\in E$, then the following optimization model is devised to determine an optimal group GLTS.

(8) $$\begin{array}{l} \min J=\sum_{k=1}^{q}\lambda_{k}\left(d({\tilde{v}}_{t_{\alpha }}^{},{\tilde{v}}_{t_{\alpha }}^{\left(k\right)})+d({\tilde{v}}_{t_{\beta }}^{},{\tilde{v}}_{t_{\beta }}^{\left(k\right)})+\ldots +d({\tilde{v}}_{t_{\gamma }}^{},{\tilde{v}}_{t_{\gamma }}^{\left(k\right)})\right)\\ s.t.\theta_{1}>0,\;\theta_{2}>0 \end{array}$$

where d(. , .) is the Euclidean distance defined by (6) and $\theta_{1},\;\theta_{2}$ are decision variables.

Solving the above nonlinear programming model yields an optimal solution denoted by $\theta_{1}^{*}$ and $\theta_{2}^{*}$. By plugging $\theta_{1}^{*}$ and $\theta_{2}^{*}$ into (3)–(5), we obtain an optimal group GLTS as

(9) $${\tilde{S}}^{*}=\left\{\langle s_{i},{\tilde{v}}_{i}^{*}\rangle |i=-\tau_{1},\ldots ,0,\ldots ,\tau_{2},{\tilde{v}}_{i}^{*}=(v_{i}^{*L},v_{i}^{*M},v_{i}^{*U})\right\}$$

where

(10) $$v_{i}^{*L}=\{\begin{array}{ll} {\left(1+e^{-\theta_{2}^{*}i}\right)}^{-1}, & i=-\tau_{1}\\ {\left(1+e^{-\theta_{2}^{*}(i-1)}\right)}^{-1}, & i=-\tau_{1}+1,\ldots ,-1,0\\ {\left(1+e^{-\theta_{1}^{*}(i-1)}\right)}^{-1}, & i=1,2,\ldots ,\tau_{2} \end{array}$$

(11) $$v_{i}^{*M}=\{\begin{array}{ll} {\left(1+e^{-\theta_{2}^{*}i}\right)}^{-1}, & i=-\tau_{1},\ldots ,-1\\ 0.5, & i=0\\ {\left(1+e^{-\theta_{1}^{*}i}\right)}^{-1}, & i=1,2,\ldots ,\tau_{2} \end{array}$$

(12) $$v_{i}^{*U}=\{\begin{array}{ll} {\left(1+e^{-\theta_{2}^{*}(i+1)}\right)}^{-1}, & i=-\tau_{1},\ldots ,-1\\ {\left(1+e^{-\theta_{1}^{*}(i+1)}\right)}^{-1}, & i=0,1,\ldots ,\tau_{2}-1\\ {\left(1+e^{-\theta_{1}^{*}i}\right)}^{-1},\; & i=\tau_{2} \end{array}$$

We can see from (9)–(12) that the optimal group GLTS ${\tilde{S}}^{*}$ captures and synthesizes individual decision makers’ risk preferences. If $\theta_{1}^{*}>\theta_{2}^{*}$, then the expert group prefers to make a risk-seeking decision. If $\theta_{1}^{*}=\theta_{2}^{*}$, then the expert group prefers to obtain a neutral-risk decision result. If $\theta_{1}^{*}<\theta_{2}^{*}$, then the expert group prefers to make a risk-aversion decision.

**Example 1.**  *Consider the LTS* S *given by (1). Three decision makers *$e_{1},e_{2}$ *and* $e_{3}$ *provide their expected triangular fuzzy semantic information as follows.*

$${\tilde{V}}^{\left(1\right)}=\left\{{\tilde{v}}_{-2}^{\left(1\right)}=(0.2,0.3,0.4),{\tilde{v}}_{0}^{\left(1\right)}=(0.6,0.65,0.75),{\tilde{v}}_{1}^{\left(1\right)}=(0.65,0.75,0.8)\right\},$$

$${\tilde{V}}^{\left(2\right)}=\left\{{\tilde{v}}_{-3}^{\left(2\right)}=(0.25,0.25,0.35),{\tilde{v}}_{1}^{\left(2\right)}=(0.6,0.7,0.75),{\tilde{v}}_{2}^{\left(2\right)}=(0.7,0.75,0.85)\right\},$$

$${\tilde{V}}^{\left(3\right)}=\left\{\begin{array}{l} {\tilde{v}}_{-3}^{\left(3\right)}=(0.18,0.18,0.28),{\tilde{v}}_{-2}^{\left(3\right)}=(0.18,0.28,0.38),{\tilde{v}}_{-1}^{\left(3\right)}=(0.28,0.38,0.48),\\ {\tilde{v}}_{0}^{\left(3\right)}=(0.38,0.48,0.58),{\tilde{v}}_{1}^{\left(3\right)}=(0.48,0.58,0.68),{\tilde{v}}_{2}^{\left(3\right)}=(0.58,0.68,0.78),\\ {\tilde{v}}_{3}^{\left(3\right)}=(0.68,0.78,0.78) \end{array}\right\}.$$

Assume that the important weights of the three decision makers are 0.4, 0.4 and 0.2 respectively. By solving the optimization model (8), we obtain an optimal solution of $\theta_{1}^{*}=0.5114$ and $\theta_{2}^{*}=0.4397$. Thus, as per (9)–(12), an optimal group GLTS is determined as

(13) $${\tilde{S}}_{1}^{*}=\left\{\begin{array}{l} \langle s_{-3},(0.2110,0.2110,0.2933)\rangle ,\langle s_{-2},(0.2110,0.2933,0.3918)\rangle ,\\ \langle s_{-1},(0.2933,0.3918,0.5000)\rangle ,\langle s_{0},(0.3918,0.5000,0.6251)\rangle ,\\ \langle s_{1},(0.5000,0.6251,0.7355)\rangle ,\langle s_{2},(0.6251,0.7355,0.8226)\rangle ,\\ \langle s_{3},(0.7355,0.8226,0.8226)\rangle \end{array}\right\}$$

**Figure 1:**
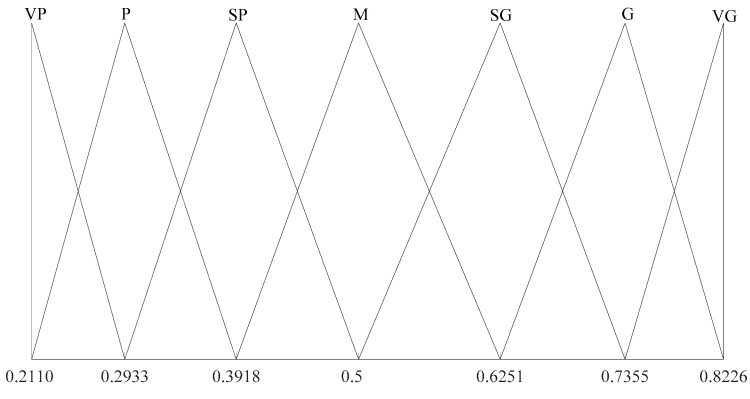
Distribution of the semantic values of the generalized linguistic term sets (GLTS) ${\tilde{S}}_{1}^{*}$.

Obviously, $\theta_{1}^{*}>\theta_{2}^{*}$, indicating that ${\tilde{S}}^{*}$ is a risk-seeking GLTS. The distribution of the semantic values of ${\tilde{S}}^{*}$ is shown in Figure 1 , where VP, P, SP, M, SG, G and VG are defined in (1). It is easy to verify that $d({\tilde{v}}_{-i}^{},{\tilde{v}}_{0}^{})\neq d({\tilde{v}}_{i}^{*},{\tilde{v}}_{0}^{*})$(i=1,2,3) and there exists i∈{−2, −1, 0, 1, 2} satisfying $d({\tilde{v}}_{i}^{*},{\tilde{v}}_{i+1}^{*})\neq d({\tilde{v}}_{i-1}^{*},{\tilde{v}}_{i}^{*})$. Hence, the distribution of the semantic values of ${\tilde{S}}^{*}$ is asymmetrical and non-uniform.

## 4. An Approach to Linguistic MAGDM with Risk Preferences and Incomplete Weight Information

This section uses the triangular fuzzy weighted average based aggregation method to fuse individual linguistic evaluations into a group triangular fuzzy evaluation and develops a linear program to obtain optimal attribute weights. A procedure is also devised for solving linguistic MAGDM problems with risk preferences and incomplete weight information.

Once a group GLTS ${\tilde{S}}^{*}$ is determined, each linguistic-term-based decision matrix $R_{k}={\left(s_{r_{ijk}}\right)}_{n\times m}$(k=1,2,…,q) can be transformed into a triangular fuzzy decision matrix denoted by

(14) $${\tilde{D}}_{k}={\left({\tilde{d}}_{ijk}\right)}_{n\times m}={\left(\left(d_{ijk}^{L},d_{ijk}^{M},d_{ijk}^{U}\right)\right)}_{n\times m},\;k=1,2,\ldots ,q$$

where

(15) $${\tilde{d}}_{ijk}={\tilde{v}}_{r_{ijk}}^{*}=(v_{r_{ijk}}^{*L},v_{r_{ijk}}^{*M},v_{r_{ijk}}^{*U}),\;i=1,2,\ldots ,n,\;j=1,2,\ldots ,m,\;k=1,2,\ldots ,q$$

Based on the triangular fuzzy decision matrices ${\tilde{D}}_{k}$(k=1,2,…,q), using the triangular fuzzy weighted average operator together with the decision makers’ weight vector $\lambda ={\left(\lambda_{1},\lambda_{2},\ldots ,\lambda_{q}\right)}^{T}$ yields a group triangular fuzzy decision matrix as

(16) $$\tilde{G}={\left({\tilde{g}}_{ij}\right)}_{n\times m}={\left(\left(g_{ij}^{L},g_{ij}^{M},g_{ij}^{U}\right)\right)}_{n\times m}$$

where

(17) $${\tilde{g}}_{ij}=\sum_{k=1}^{q}\lambda_{k}{\tilde{d}}_{ijk}=\left(\sum_{k=1}^{q}\lambda_{k}v_{r_{ijk}}^{*L},\sum_{k=1}^{q}\lambda_{k}v_{r_{ijk}}^{*M},\sum_{k=1}^{q}\lambda_{k}v_{r_{ijk}}^{*U}\right)$$

In MAGDM problems, it is clear that different attribute weights reflect their importance in selecting the best alternative and ranking alternatives. Let $w={\left(w_{1},w_{2},\ldots ,w_{m}\right)}^{T}$ be the attribute crisp weight vector, where $\sum_{j=1}^{m}w_{j}=1$ and $w_{j}\geq 0$, j=1,2,…,m. If the attribute weights in w are completely known, then from the group decision matrix $\tilde{G}$, a group overall evaluation value of alternative $x_{i}$ is determined as

(18) $$\begin{array}{l} {\tilde{d}}_{i}^{\left(G\right)}=\sum_{j=1}^{m}w_{j}{\tilde{g}}_{ij}=\left(\sum_{j=1}^{m}g_{ij}^{L}w_{j},\sum_{j=1}^{m}g_{ij}^{M}w_{j},\sum_{j=1}^{m}g_{ij}^{U}w_{j}\right)\\ =\left(\sum_{j=1}^{m}\left(w_{j}\sum_{k=1}^{q}\lambda_{k}v_{r_{ijk}}^{*L}\right),\sum_{j=1}^{m}\left(w_{j}\sum_{k=1}^{q}\lambda_{k}v_{r_{ijk}}^{*M}\right),\sum_{j=1}^{m}\left(w_{j}\sum_{k=1}^{q}\lambda_{k}v_{r_{ijk}}^{*U}\right)\right) \end{array}$$

for i=1,2,…,n.

In reality, it is often difficult for decision makers to offer exact values for attribute weights due to the complexity of practical decision cases and the limitation of the decision makers’ knowledge. Thus, the attribute weight information may be incomplete or partially known, which can be characterized by a nonempty subset Ω of all combinations of the following five forms.

(i) A weak ranking: $\{w_{i}\geq w_{j}\},i\neq j$;
(ii) A strict ranking: $\{w_{i}-w_{j}\geq \varepsilon_{ij}\},i\neq j$, where $\varepsilon_{ij}>0$;
(iii) An interval form: $\left\{\alpha_{j}\leq w_{j}\leq \alpha_{j}+\varepsilon_{j}\right\}$, where $0\leq \alpha_{j}<\alpha_{j}+\varepsilon_{j}\leq 1$;
(iv) A ranking with multiples: $\left\{w_{i}\geq \beta_{ij}w_{j}\right\}$, where $0\leq \beta_{ij}\leq 1,i\neq j;$ and
(v) A ranking of deviations: $\left\{w_{i}-w_{j}\geq w_{k}-w_{l}\right\}$, where i≠j≠k≠l.

Since the value ${\tilde{d}}_{i}^{\left(G\right)}$ defined by (18) indicates the overall evaluation of alternative $x_{i}$, the larger the triangular fuzzy number ${\tilde{d}}_{i}^{\left(G\right)}$, the better the alternative $x_{i}$ is. Thus, as per the score of a triangular fuzzy number given by (7), we should find a weight vector $w={\left(w_{1},w_{2},\cdots ,w_{m}\right)}^{T}$ such that $S({\tilde{d}}_{i}^{\left(G\right)})$ is maximized for all i=1,2,…,n. Therefore, the following multi-objective optimization model is established to determine attribute weights.

(19) $$\begin{array}{l} \max J_{x_{i}}=S({\tilde{d}}_{i}^{\left(G\right)})\;i=1,2,\ldots ,n\\ s.t.\{\begin{array}{ll} 0\leq w_{j}\leq 1, & j=1,2,\ldots ,m\\ \sum_{j=1}^{m}w_{j}=1, & \\ w={\left(w_{1},w_{2},\cdots ,w_{m}\right)}^{T}\in \Omega . & \end{array} \end{array}$$

Since each alternative $x_{i}\in X$ is a feasible and non-inferior alternative and the maximization problems $\max J_{x_{i}}=S({\tilde{d}}_{i}^{\left(G\right)})$ (i=1,2,…,n) have the same constraint conditions, the multi-objective optimization model (19) can be converted into the following aggregated optimization model by setting the same important weight for each goal $J_{x_{i}}$(i=1,2,…,n).

(20) $$\begin{array}{l} \max J=\frac{1}{n}\sum_{i=1}^{n}S({\tilde{d}}_{i}^{\left(G\right)})\\ s.t.\{\begin{array}{ll} 0\leq w_{j}\leq 1, & j=1,2,\ldots ,m\\ \sum_{j=1}^{m}w_{j}=1, & \\ w={\left(w_{1},w_{2},\cdots ,w_{m}\right)}^{T}\in \Omega . & \end{array} \end{array}$$

As per (7) and (18), the optimization model (20) can be equivalently rewritten as the following linear program.

(21) $$\begin{array}{l} \max J=\frac{1}{4n}\sum_{i=1}^{n}\sum_{j=1}^{m}\left(g_{ij}^{L}+2g_{ij}^{M}+g_{ij}^{U}\right)w_{j}\\ s.t.\{\begin{array}{ll} 0\leq w_{j}\leq 1, & j=1,2,\ldots ,m\\ \sum_{j=1}^{m}w_{j}=1, & \\ w={\left(w_{1},w_{2},\cdots ,w_{m}\right)}^{T}\in \Omega . & \end{array} \end{array}$$

where $w_{j}$ is a decision variable for all j=1,2,…,m.

By solving the linear program (21), we obtain an optimal attribute weight vector denoted by $w^{*}={\left(w_{1}^{*},w_{2}^{*},\cdots ,w_{m}^{*}\right)}^{T}$.

Substituting $w^{*}$ into (18) yields an optimal group overall evaluation value of alternative $x_{i}$ as

(22) $$\begin{array}{l} {\tilde{d}}_{i}^{\left(*G\right)}=\left(\sum_{j=1}^{m}g_{ij}^{L}w_{j}^{*},\sum_{j=1}^{m}g_{ij}^{M}w_{j}^{*},\sum_{j=1}^{m}g_{ij}^{U}w_{j}^{*}\right)\\ =\left(\sum_{j=1}^{m}\left(w_{j}^{*}\sum_{k=1}^{q}\lambda_{k}v_{r_{ijk}}^{*L}\right),\sum_{j=1}^{m}\left(w_{j}^{*}\sum_{k=1}^{q}\lambda_{k}v_{r_{ijk}}^{*M}\right),\sum_{j=1}^{m}\left(w_{j}^{*}\sum_{k=1}^{q}\lambda_{k}v_{r_{ijk}}^{*U}\right)\right) \end{array}$$

Based on the aforementioned analysis, a procedure is now developed for linguistic MAGDM with risk preferences and incomplete attribute weigh information.

### Procedure

Step 1: Each decision maker $e_{k}\in E$ (k=1,2,…,q) adopts linguistic terms in S to evaluate alternatives in *X* with respect to each attribute in A, which are given by a decision matrix $R_{k}={\left(s_{r_{ijk}}\right)}_{n\times m},$ and provides his/her risk preference information ${\tilde{V}}^{\left(k\right)}$.

Step 2: Obtain an optimal group GLTS ${\tilde{S}}^{*}$ by solving the optimization model (8) and using (9)–(12).

Step 3: Transform the linguistic-term-based decision matrix $R_{k}$ into a triangular fuzzy decision matrix ${\tilde{D}}_{k}$ as per (14) and (15) for each k=1,2,…,q.

Step 4: Aggregate individual decision matrix ${\tilde{D}}_{k}$ (k=1,2,…,q) into a group triangular fuzzy decision matrix $\tilde{G}={\left({\tilde{g}}_{ij}\right)}_{n\times m}={\left(\left(g_{ij}^{L},g_{ij}^{M},g_{ij}^{U}\right)\right)}_{n\times m}$ according to (17).

Step 5: Determine optimal attribute weights by solving the linear program (21).

Step 6: Employ (22) to obtain optimal group overall evaluation values ${\tilde{d}}_{i}^{\left(*G\right)}$ (i=1,2,…,n) for all alternatives in X.

Step 7: Use (7) to calculate scores $S({\tilde{d}}_{i}^{\left(*G\right)})$(i=1,2,…,n).

Step 8: Obtain a ranking order of all alternatives in terms of the decreasing order of the scores $S({\tilde{d}}_{i}^{\left(*G\right)})$(i=1,2,…,n), and $x_{i}≻x_{j}$ is employed to express that alternative $x_{i}$ is preferred to $x_{j}$.

## 5. A Case Study of the Low-Carbon Tourism Destination Selection Problem

This section applies the proposed linguistic MAGDM model to examine a low-carbon tourism destination selection problem.

With the continuing advocacy and promotion of the Chinese government, many tourism destinations have been developed to reduce carbon emissions and save energy. Moreover, many tourists have recognized the importance of low-carbon tourism for environmental protection. In order to find a good balance between the enjoyment of a trip and carbon emission reduction, it is crucial for tourists to compare and evaluate some known low-carbon tourism destinations, and then choose the best one(s) from these options. Generally speaking, this evaluation and selection process is based on several criteria or attributes. In this case study, the attributes consist of the following five aspects:

(i) $a_{1}$: Low-carbon transportation, low-energy consumption vehicles and pick-up and drop-off services as reflected in connecting different scenic sites and reaching the destination.
(ii) $a_{2}$: Food service including green food, a low-carbon environment and low-energy waste handling mechanisms.
(iii) $a_{3}$: Hotels and accommodation, as reflected in green-material labels, low-carbon facilities and a low-carbon environment and education management.
(iv) $a_{4}$: Consumption satisfaction, as reflected in the service cost of travel agencies, ticket prices for scenic sites and the cost of accommodation.
(v) $a_{5}$: Attraction and impact of scenic sites, including low-carbon customer service and low-carbon management and control.

Without loss of generality, assume that three tourists (i.e., decision makers) $e_{1},\;e_{2}$ and $e_{3}$ want to go on a low-carbon trip and their importance weights are 0.4, 0.4 and 0.2, respectively, i.e., $\lambda ={\left(0.4,0.4,0.2\right)}^{T}$. After preliminary screening there are four low-carbon tourism destinations $x_{1},\;x_{2},\;x_{3}$ and $x_{4}$ as the alternatives. Based on the LTS *S* given by (1), the three tourists provide their linguistic evaluations for the four tourism destinations with respect to each attribute $a_{j}$(j=1,2,…,n). The three tourists’ linguistic evaluations are shown in Table 1, Table 2 and Table 3, respectively. 

Based on the expectations of semantic scale values of linguistic terms in S, the expected triangular fuzzy semantic values for the three tourists are as follows:

$${\tilde{V}}^{\left(1\right)}=\left\{{\tilde{v}}_{-2}^{\left(1\right)}=(0.15,0.25,0.35),{\tilde{v}}_{1}^{\left(1\right)}=(0.55,0.65,0.75),{\tilde{v}}_{2}^{\left(1\right)}=(0.65,0.75,0.85)\right\}\;{\tilde{V}}^{\left(2\right)}=\left\{{\tilde{v}}_{-2}^{\left(2\right)}=(0.25,0.35,0.45),{\tilde{v}}_{1}^{\left(2\right)}=(0.6,0.7,0.8),{\tilde{v}}_{2}^{\left(2\right)}=(0.7,0.8,0.9)\right\},$$

$${\tilde{V}}^{\left(3\right)}=\left\{\begin{array}{l} {\tilde{v}}_{-3}^{\left(3\right)}=(0.2,0.2,0.3),{\tilde{v}}_{-2}^{\left(3\right)}=(0.2,0.3,0.4),{\tilde{v}}_{-1}^{\left(3\right)}=(0.3,0.4,0.5),{\tilde{v}}_{0}^{\left(3\right)}=(0.4,0.5,0.6),\\ {\tilde{v}}_{1}^{\left(3\right)}=(0.5,0.6,0.7),{\tilde{v}}_{2}^{\left(3\right)}=(0.6,0.7,0.8),{\tilde{v}}_{3}^{\left(3\right)}=(0.7,0.8,0.8) \end{array}\right\}$$

Solving the nonlinear programming model (8) yields an optimal solution of $\theta_{1}^{*}=0.5668,\theta_{2}^{*}=0.4417$. By (9)–(12), an optimal group GLTS is obtained as

$${\tilde{S}}_{2}^{*}=\left\{\begin{array}{l} \langle s_{-3},(0.2100,0.2100,0.2925)\rangle ,\langle s_{-2},(0.2100,0.2925,0.3913)\rangle ,\\ \langle s_{-1},(0.2925,0.3913,0.5000)\rangle ,\langle s_{0},(0.3913,0.5000,0.6380)\rangle ,\\ \langle s_{1},(0.5000,0.6380,0.7565)\rangle ,\langle s_{2},(0.6380,0.7565,0.8456)\rangle ,\\ \langle s_{3},(0.7565,0.8456,0.8456)\rangle \end{array}\right\}$$

According to (14) and (15), the decision matrices $R_{k}$ (k=1,2,3) are converted to triangular fuzzy decision matrices ${\tilde{D}}_{k}$ (k=1,2,3), which are shown in Table 4, Table 5 and Table 6, respectively. As per (17), a group triangular fuzzy decision matrix $\tilde{G}$ is determined as listed in Table 7.

Assume further that the three tourists provide their incomplete attribute weight information as

$$\Omega =\left\{w_{3}-w_{1}\geq w_{2}-w_{4},w_{1}\geq 0.8w_{4},0.1\leq w_{3}\leq 0.2,0.15\leq w_{5}\leq 0.25\right\}$$

Thus, based on (21), a linear program is established as

(23) $$\begin{array}{l} \max J=0.5813w_{1}+0.71w_{2}+0.6214w_{3}+0.5666w_{4}+0.6594w_{5}\\ s.t.\{\begin{array}{l} 0\leq w_{1}\leq 1,0\leq w_{2}\leq 1,0\leq w_{3}\leq 1,0\leq w_{4}\leq 1,\;0\leq w_{5}\leq 1,\\ \sum_{j=1}^{5}w_{j}=1,\\ w_{3}-w_{1}\geq w_{2}-w_{4},w_{1}\geq 0.8w_{4},0.1\leq w_{3}\leq 0.2,0.15\leq w_{5}\leq 0.25. \end{array} \end{array}$$

Solving (23) yields an optimal attribute weight vector as $w=(0.1400,0.2350,0.2,0.1750,0.2500{)}^{T}$. 

As per (22), the optimal group overall evaluation values are determined as follows.

$${\tilde{d}}_{1}^{\left(*G\right)}=(0.5511,0.6622,0.7451),{\tilde{d}}_{2}^{\left(*G\right)}=(0.5396,0.6397,0.7162),\;{\tilde{d}}_{3}^{\left(*G\right)}=(0.5220,0.6332,0.7260),{\tilde{d}}_{4}^{\left(*G\right)}=(0.5114,0.6319,0.7377).$$

Using (7), we obtain $S({\tilde{d}}_{1}^{\left(*G\right)})=0.6451$, $S({\tilde{d}}_{2}^{\left(*G\right)})=0.6338$, $S({\tilde{d}}_{3}^{\left(*G\right)})=0.6286$ and $S({\tilde{d}}_{4}^{\left(*G\right)})=0.6282$.

Since $S({\tilde{d}}_{1}^{\left(*G\right)})>S({\tilde{d}}_{2}^{\left(*G\right)})>S({\tilde{d}}_{3}^{\left(*G\right)})>S({\tilde{d}}_{4}^{\left(*G\right)})$, the four low-carbon tourism destinations are ranked as $x_{1}≻x_{2}≻x_{3}≻x_{4}$ and thus, $x_{1}$ is the best low-carbon tourism destination.

Next, a study is made to compare the attribute weight vector and the ranking order obtained from the proposed model herein with the results derived from the 2-tuple linguistic based approaches by Wei [12,14] and Ju [16].

Wei [12] first converted individual linguistic-term-based decision matrices to 2-tuple linguistic decision matrices, which are then aggregated into a group decision matrix. Based on the TOPSIS method, Wei [12] developed an optimization model to obtain an optimal attribute weight vector. For this case study, using this optimization model yields the optimal attribute weight vector as $w=(0.3333,\;0,\;0.1,\;0.4167,\;0.15{)}^{T}$ and a ranking order of the four low-carbon tourism destinations is determined as $x_{3}≻x_{4}≻x_{1}≻x_{2}$. The results are shown in the second row in Table 8. 

Wei [14] used the maximizing deviation method to establish an optimization model for determining an optimal attribute weight vector and employed grey relational analysis to obtain a ranking order of all alternatives. Using this maximizing deviation based model generates the optimal attribute weight vector as $w=(0.425,\;0,\;0.2,\;0.225,\;0.15{)}^{T}$ and thus, a ranking order of the four low-carbon tourism destinations is obtained as $x_{3}≻x_{2}≻x_{4}≻x_{1}$. The results are listed in the third row in Table 8. 

Ju [16] aggregated individual 2-tuple linguistic decision matrices into a group decision matrix whose symbolic translation values belong to $[-\frac{1}{2(\tau_{1}+\tau_{2})},\frac{1}{2(\tau_{1}+\tau_{2})})$ and constructed a TOPSIS-based optimization model to obtain an optimal attribute weight vector. By using Ju’s approach [16], we obtain the optimal attribute weight vector as $w=(0.425,\;0,\;0.1,\;0.325,\;0.15{)}^{T}$ and the ranking order of the four low-carbon tourism destinations as $x_{3}≻x_{4}≻x_{1}≻x_{2}$, which are shown in the penultimate row in Table 8.

Table 8 reveals that the ranking order obtained by the proposed model in this paper differs from the results derived by the 2-tuple linguistic based approaches in [12,14,16]. This difference is mainly due to the fact that the 2-tuple linguistic based approaches [12,14,16] adopt symbolic translation models to obtain a group decision matrix and do not consider decision makers’ risk preferences for semantic scales of linguistic terms. As a result of this treatment, the importance weight of attribute $a_{2}$ is determined to be 0. In other words, when the linguistic MAGDM methods [12,14,16] are applied in solving this low-carbon tourism destination selection problem, the evaluation criterion $a_{2}$ is excluded from the consideration. It can be seen from Table 8 that by our proposed model, $a_{2}$ is determined to be a pivotal criterion for the low-carbon tourism destination selection.

## 6. Conclusions

In this paper, a nonlinear programming model has been established to obtain an optimal group GLTS based on individual risk preferences. An aggregation method has been presented to fuse individual linguistic-term-based evaluation values into a group evaluation with triangular fuzzy information. By maximizing the score of the group overall evaluation value for each alternative, a multi-objective optimization model has been devised and converted into a linear program for determining an optimal attribute weight vector. An approach has been developed for linguistic MAGDM with risk preferences and incomplete weight information. A low-carbon tourism destination selection case study has been provided to demonstrate the use of the proposed group decision-making model.

## Figures and Tables

**Table 1:** Linguistic-term-based decision matrix $R_{1}={\left(s_{r_{ij1}}\right)}_{4\times 5}$ given by $e_{1}$.

Alternative	$a_{1}$	$a_{2}$	$a_{3}$	$a_{4}$	$a_{5}$
$x_{1}$	P	G	VG	M	SG
$x_{2}$	SP	M	G	P	G
$x_{3}$	VG	G	SP	SP	M
$x_{4}$	SG	SG	M	G	M

**Table 2:** Linguistic-term-based decision matrix $R_{2}={\left(s_{r_{ij2}}\right)}_{4\times 5}$ given by $e_{2}$.

Alternative	$a_{1}$	$a_{2}$	$a_{3}$	$a_{4}$	$a_{5}$
$x_{1}$	SP	VG	SG	G	G
$x_{2}$	SG	VG	VG	VP	VG
$x_{3}$	G	VG	M	G	SG
$x_{4}$	SG	G	SP	SG	SG

**Table 3:** Linguistic-term-based decision matrix $R_{3}={\left(s_{r_{ij3}}\right)}_{4\times 5}$ given by $e_{3}$.

Alternative	$a_{1}$	$a_{2}$	$a_{3}$	$a_{4}$	$a_{5}$
$x_{1}$	M	SP	G	SG	VG
$x_{2}$	G	SG	SG	M	G
$x_{3}$	G	SG	G	G	SP
$x_{4}$	M	VG	SG	VG	G

**Table 4:** Triangular fuzzy decision matrix ${\tilde{D}}_{1}={\left({\tilde{d}}_{ij1}\right)}_{4\times 5}$.

Alternative	$a_{1}$	$a_{2}$	$a_{3}$	$a_{4}$	$a_{5}$
$x_{1}$	(0.2100,0.2925,0.3913)	(0.6380,0.7565,0.8456)	(0.7565,0.8456,0.8456)	(0.3913,0.5000,0.6380)	(0.5000,0.6380,0.7565)
$x_{2}$	(0.2925,0.3913,0.5000)	(0.3913,0.5000,0.6380)	(0.6380,0.7565,0.8456)	(0.2100,0.2925,0.3913)	(0.6380,0.7565,0.8456)
$x_{3}$	(0.7565,0.8456,0.8456)	(0.6380,0.7565,0.8456)	(0.2925,0.3913,0.5000)	(0.2925,0.3913,0.5000)	(0.3913,0.5000,0.6380)
$x_{4}$	(0.5000,0.6380,0.7565)	(0.5000,0.6380,0.7565)	(0.3913,0.5000,0.6380)	(0.6380,0.7565,0.8456)	(0.3913,0.5000,0.6380)

**Table 5:** Triangular fuzzy decision matrix ${\tilde{D}}_{2}={\left({\tilde{d}}_{ij2}\right)}_{4\times 5}$.

Alternative	$a_{1}$	$a_{2}$	$a_{3}$	$a_{4}$	$a_{5}$
$x_{1}$	(0.2925,0.3913,0.5000)	(0.7565,0.8456,0.8456)	(0.5000,0.6380,0.7565)	(0.6380,0.7565,0.8456)	(0.6380,0.7565,0.8456)
$x_{2}$	(0.5000,0.6380,0.7565)	(0.7565,0.8456,0.8456)	(0.7565,0.8456,0.8456)	(0.2100,0.2100,0.2925)	(0.7565,0.8456,0.8456)
$x_{3}$	(0.6380,0.7565,0.8456)	(0.7565,0.8456,0.8456)	(0.3913,0.5000,0.6380)	(0.6380,0.7565,0.8456)	(0.5000,0.6380,0.7565)
$x_{4}$	(0.5000,0.6380,0.7565)	(0.6380,0.7565,0.8456)	(0.2925,0.3913,0.5000)	(0.5000,0.6380,0.7565)	(0.5000,0.6380,0.7565)

**Table 6:** Triangular fuzzy decision matrix ${\tilde{D}}_{3}={\left({\tilde{d}}_{ij3}\right)}_{4\times 5}$.

Alternative	$a_{1}$	$a_{2}$	$a_{3}$	$a_{4}$	$a_{5}$
$x_{1}$	(0.3913,0.5000,0.6380)	(0.2925,0.3913,0.5000)	(0.6380,0.7565,0.8456)	(0.5000,0.6380,0.7565)	(0.7565,0.8456,0.8456)
$x_{2}$	(0.6380,0.7565,0.8456)	(0.5000,0.6380,0.7565)	(0.5000,0.6380,0.7565)	(0.3913,0.5000,0.6380)	(0.6380,0.7565,0.8456)
$x_{3}$	(0.6380,0.7565,0.8456)	(0.5000,0.6380,0.7565)	(0.6380,0.7565,0.8456)	(0.6380,0.7565,0.8456)	(0.2925,0.3913,0.5000)
$x_{4}$	(0.3913,0.5000,0.6380)	(0.7565,0.8456,0.8456)	(0.5000,0.6380,0.7565)	(0.7565,0.8456,0.8456)	(0.6380,0.7565,0.8456)

**Table 7:** Group triangular fuzzy decision matrix $\tilde{G}={\left({\tilde{g}}_{ij}\right)}_{4\times 5}={\left(\left(g_{ij}^{L},g_{ij}^{M},g_{ij}^{U}\right)\right)}_{4\times 5}$.

Alternative	$A_{1}$	$A_{2}$	$A_{3}$	$A_{4}$	$A_{5}$
$X_{1}$	(0.2793,0.3735,0.4841)	(0.6163,0.7191,0.7765)	(0.6302,0.7447,0.8100)	(0.5117,0.6302,0.7447)	(0.6065,0.7269,0.8100)
$X_{2}$	(0.4446,0.5630,0.6717)	(0.5591,0.6658,0.7447)	(0.6578,0.7684,0.8278)	(0.2463,0.3010,0.4011)	(0.6854,0.7921,0.8456)
$X_{3}$	(0.6854,0.7921,0.8456)	(0.6578,0.7684,0.8278)	(0.4011,0.5078,0.6243)	(0.4998,0.6104,0.7074)	(0.4150,0.5335,0.6578)
$X_{4}$	(0.4783,0.6104,0.7328)	(0.6065,0.7269,0.8100)	(0.3735,0.4841,0.6065)	(0.6065,0.7269,0.8100)	(0.4841,0.6065,0.7269)

**Table 8:** A comparative study for attribute weight vectors and ranking results obtained from different models.

Model	Ref.	Attribute Weight Vector	Ranking Result
(M-3) and (20)	Wei [12]	$w={\left(0.3333,\;0,\;0.1,\;0.4167,\;0.15\right)}^{T}$	$x_{3}≻x_{4}≻x_{1}≻x_{2}$
(M-2) and (11)–(19)	Wei [14]	$w={\left(0.425,\;0,\;0.2,\;0.225,\;0.15\right)}^{T}$	$x_{3}≻x_{2}≻x_{4}≻x_{1}$
(M-5) and (8)	Ju [16]	$w={\left(0.425,\;0,\;0.1,\;0.325,\;0.15\right)}^{T}$	$x_{3}≻x_{4}≻x_{1}≻x_{2}$
(21) and (22)	This paper	$w={\left(0.14,\;0.235,\;0.2,\;0.175,\;0.25\right)}^{T}$	$x_{1}≻x_{2}≻x_{3}≻x_{4}$
